# Potential of Non-Coding RNA as Biomarkers for Progressive Supranuclear Palsy

**DOI:** 10.3390/ijms232314554

**Published:** 2022-11-22

**Authors:** Fabio A. Simoes, Greig Joilin, Oliver Peters, Luisa-Sophie Schneider, Josef Priller, Eike Jakob Spruth, Ina Vogt, Okka Kimmich, Annika Spottke, Daniel C. Hoffmann, Björn Falkenburger, Moritz Brandt, Johannes Prudlo, Kathrin Brockmann, Franca Laura Fries, James B. Rowe, Alistair Church, Gesine Respondek, Sarah F. Newbury, P. Nigel Leigh, Huw R. Morris, Günter U. Höglinger, Majid Hafezparast

**Affiliations:** 1 School of Life Sciences, University of Sussex, Brighton BN1 9QG, UK; 2 German Center for Neurodegenerative Diseases (DZNE), Germany; 3 Department of Psychiatry, Charité-Universitätsmedizin Berlin, 12203 Berlin, Germany; 4 Department of Psychiatry and Psychotherapy, Charité, 10117 Berlin, Germany; 5 Department of Psychiatry and Psychotherapy, Klinikum Rechts der Isar, Technical University Munich, 81675 Munich, Germany; 6 Department of Neurology, University of Bonn, Bonn 53127, Germany; 7 Department of Neurology, Technische Universität Dresden, 01307 Dresden, Germany; 8 Department of Neurology, Rostock University Medical Center, 18147 Rostock, Germany; 9 Department for Neurodegenerative Diseases, Hertie Institute for Clinical Brain Research, University of Tübingen, 72076 Tübingen, Germany; 10 Department of Clinical Neurosciences and Cambridge University Hospitals NHS Trust, University of Cambridge, Cambridge CB2 0QQ, UK; 11 Medical Research Council Cognition and Brain Sciences Unit, Cambridge CB2 7EF, UK; 12 Department of Neurology, Royal Gwent Hospital, Newport NP20 2UB, UK; 13 Department of Neurology, Technische Universität München, 81377 Munich, Germany; 14 Brighton and Sussex Medical School, Brighton BN1 9QG, UK; 15 Department of Clinical and Movement Neurosciences, UCL Queen Square Institute of Neurology, University College London, London WC1N 3BG, UK

**Keywords:** progressive supranuclear palsy, PSP, biomarker, non-coding RNA, RNA-seq

## Abstract

Objective markers for the neurodegenerative disorder progressive supranuclear palsy (PSP) are needed to provide a timely diagnosis with greater certainty. Non-coding RNA (ncRNA), including microRNA, piwi-interacting RNA, and transfer RNA, are good candidate markers in other neurodegenerative diseases, but have not been investigated in PSP. Therefore, as proof of principle, we sought to identify whether they were dysregulated in matched serum and cerebrospinal fluid (CSF) samples of patients with PSP. Small RNA-seq was undertaken on serum and CSF samples from healthy controls (n = 20) and patients with PSP (n = 31) in two cohorts, with reverse transcription-quantitative PCR (RT-qPCR) to confirm their dysregulation. Using RT-qPCR, we found in serum significant down-regulation in hsa-miR-92a-3p, hsa-miR-626, hsa-piR-31068, and tRNA-ValCAC. In CSF, both hsa-let-7a-5p and hsa-piR-31068 showed significant up-regulation, consistent with their changes observed in the RNA-seq results. Interestingly, we saw no correlation in the expression of hsa-piR-31068 within our matched serum and CSF samples, suggesting there is no common dysregulatory mechanism between the two biofluids. While these changes were in a small cohort of samples, we have provided novel evidence that ncRNA in biofluids could be possible diagnostic biomarkers for PSP and further work will help to expand this potential.

## 1. Introduction

Progressive supranuclear palsy (PSP) describes a spectrum of fatal motor and behavioural syndromes which are characterised by the neuronal and glial accumulation of 4 repeat-domain containing isoforms of the microtubule associated protein tau (4R-tau). The classical presentation of the disease is known as Richardson’s syndrome (PSP-RS) and has a prevalence in people aged between 70–74 in the United Kingdom (UK) of approximately 10.8 cases per 100,000 [1]. However, a study based in Japan, which took into account the other PSP phenotypes increased the prevalence to 18 per 100,000 across all ages [2].

PSP was long diagnosed using the National Institute of Neurological Disorders/Society for PSP (NINDS-SPSP) diagnostic criteria [3] based on vertical supranuclear gaze palsy, postural instability, and falls early in the clinical course. However, with further identification and recognition of the subtypes of PSP, the Movement Disorder Society (MDS) criteria [4] provide a new diagnostic framework, which also include cognitive changes, PSP-parkinsonism, and progressive gait freezing (PSP-PGF). However, early *ante-mortem* diagnosis remains difficult with there often being a delay of three to four years after symptom onset until a firm diagnosis is established, with nearly half of patients initially misdiagnosed with Parkinson’s disease (PD) [5,6], and approximately 10% of cases are found to have an alternative diagnosis [7]. Additionally, while most variant forms of PSP eventually display some or all of the phenotypes associated with PSP-RS [8], some variant forms of the disease are not diagnosed until post-mortem examination [4,8]. As such, robust biological markers or biomarkers to aid in the diagnosis of PSP are required. Not only will this help with differentiating between early PSP and other parkinsonian disorders, but it will aid in clinical trials for enrolment [9].

There is a range of options for biomarkers, ranging from structural, neurophysiological, or molecular measures [8]. In addition to being accurate, it is important that biomarkers be minimally invasive to obtain, simple to undertake, and time efficient. It is for this reason that biofluids, such as blood and CSF, are our primary target as potential biomarkers for PSP. While there is a range of molecules in these biofluids including DNA, and protein, some of which have been suggested as biomarkers [10,11,12], there is a growing interest in the non-coding RNA (ncRNA) class called microRNA (miRNA). MiRNAs are short non-coding single stranded RNAs which are involved in the regulation of gene expression and are known to have relatively simple structures, exhibit increased stability from RNase degradation and freeze–thaw cycles, and are easy to profile [13,14].

Indeed, miRNAs have been previously linked to PSP, with one study investigating miRNA biomarkers for PD finding some indications that miR-626 and miR-505-3p may be dysregulated in plasma in only five PSP samples [15]. Additionally, increased expression of miRNAs such as miR-147a and miR-518e-3p were reported in the forebrain of PSP patients [16], while another study found a reduction in miR-132 in human temporal cortex tissue which could contribute to the tau pathology in PSP [17]. In both of these studies, the miRNAs interacted with gene targets dysregulated in PSP suggesting they may contribute to the pathology of the disease. Additionally, in the neurodegenerative disease amyotrophic lateral sclerosis or motor neuron disease (ALS/MND), the dysregulation of muscle-enriched hsa-miR-206 in the serum of ALS patients is thought to reflect the pathology occurring in the muscle [18,19,20,21,22]. It is possible that the dysregulation of these miRNA in PSP brain tissue could be reflected in peripheral biofluids such as serum and CSF, with additional targets providing further insight into the disease pathology. Together when combined with other miRNA, they could be used to help differentiate between the different Parkinsonian disorders and provide more timely and specific diagnosis [23].

MiRNA are not the only small RNA present in biofluids, with other species including piwi-interacting RNA (piRNA) and transfer RNA (tRNA) fragments [24,25,26]. With the development of next generation RNA sequencing, we are now able to detect these additional species along with miRNA as demonstrated in our previous work where we found different types of ncRNA in the serum of patients with ALS as potential disease diagnosis tools [18]. Therefore, we hypothesized that ncRNAs, including miRNA, could also be dysregulated in PSP and that this may be reflected through their presence in serum and CSF, and could be used as diagnostic biomarkers. We therefore carried out an RNA-seq screen on serum and CSF samples followed by TaqMan RT-qPCR to validate potential biomarker candidates. We provide evidence that there is dysregulation of ncRNA in both serum and CSF of PSP patients. While there was limited validation of differential expression of the ncRNA, there may be potential dysregulated ncRNA in biofluids in PSP patients that could be used to aid in diagnosis.

## 2. Clinical Significance

- In this current study, we investigated the expression of non-coding RNA transcripts in the serum and cerebrospinal fluid of PSP patients compared to healthy controls to identify diagnostic biomarkers.
- RNA-seq identified dysregulated non-coding RNA transcripts and have confirmed with RT-qPCR that four ncRNA that were changed in serum and two ncRNA that were changed in CSF.
- We conclude that ncRNA in biofluids could be used as potential diagnostic biomarkers for PSP.

## 3. Results

### 3.1. Small RNA-Seq Shows Dysregulation of Non-Coding RNA in PSP Patients

We sought to identify dysregulated ncRNA in PSP patients that could be used as biomarkers. To do this, we undertook next generation RNA-seq to profile all small ncRNA in the serum and CSF of healthy controls and PSP patients using our established protocols [18]. This included combining our samples into age- and sex-matched pools following RNA extraction but prior to library creation to increase the signal to noise ratio, which has allowed us to identify consistently dysregulated ncRNA previously. For PSP patient pools, we pooled based on whether they had possible or probable diagnosis of PSP. Additionally, pools contained matched samples between serum and CSF.

**Figure 1:**
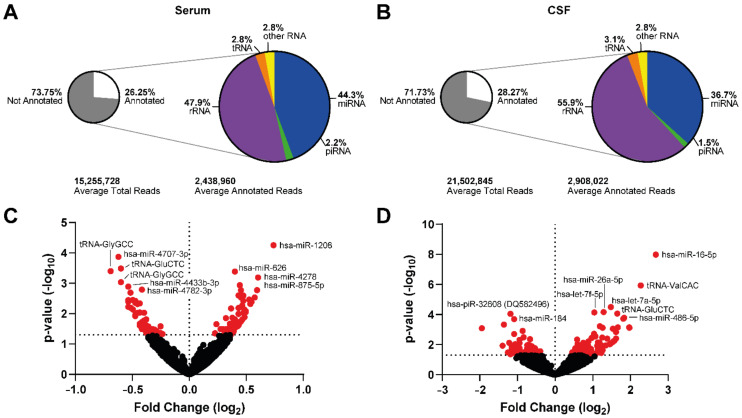
RNA-seq shows ncRNA may be dysregulated in the serum and CSF from PSP patients compared to healthy controls. Summary of the number of aligned reads that were annotated and the ncRNA species they aligned to in serum (**A**) and CSF (**B**) with volcano plots of ncRNA dysregulated in serum (**C**) and CSF (**D**). Dots in red were significantly dysregulated compared to healthy control, with the ten most significantly dysregulated labelled.

Using the Illumina NextSeq, we were able to on average generate 29.3 million reads per sample pool for the serum. Using the analysis provided on the Qiagen GeneGlobe platform, removing those reads that were too short or had no adapters, we had on average 15.3 million reads per sample pool. On average, 4.9 million reads aligned to the human genome (hg38), of which 2.4 million reads were annotated for ncRNA (Figure 1A). For CSF, 36.6 million reads per sample pool were generated on average where 21.5 million per sample pool passed post-processing, 6.1 million reads aligned to hg38 of which 2.9 million reads were annotated (Figure 1B). For both, the majority of reads were annotated to ribosomal RNA (rRNA; serum: 55.9%; CSF 47.9%) and miRNA (serum: 36.7, CSF: 44.3%), with smaller amounts aligning to piRNA and transfer RNA (tRNA) fragments (Figure 1A,B). The high levels of rRNA was likely due to degraded sequences, but it is unlikely to have biased our data due to the small percentage of raw reads annotated to rRNA.

Comparing the ncRNA expression in our PSP and control samples in the serum and CSF, we found that there was significant dysregulation across a range of species. In serum, 125 ncRNA were significantly dysregulated between PSP and control (*p* < 0.05), with 67 up-regulated and 58 down-regulated (Figure 1C). The majority of these were miRNA (106 species) with three piRNA and 16 tRNA changed. However, the expression counts of a majority of these were low, with only 23 showing at least 100 reads on average across all samples (14 up-regulated, nine down-regulated). Of these, 20 were miRNA species and three were piRNA.

In CSF, there was a similar number, with 134 significantly dysregulated (*p* < 0.05) with 71 up-regulated, and 63 down-regulated (Figure 1D). For CSF, there was slightly more diversity in the range of ncRNA types dysregulated, with 113 miRNA species, seven piRNA, 12 tRNA, one rRNA, and one snRNA. As for serum, only 35 ncRNA showed expression levels above 100 reads on average across samples (25 up-regulated, ten down-regulated). Four tRNA species, one piRNA, and 30 miRNA made up this group.

Comparing the serum and CSF, there were only 9 that were significantly dysregulated in both fluids, with four showing dysregulation in the same direction (hsa-miR-145-5p, hsa-miR-148-3p, hsa-miR-1247-3p, and hsa-miR-6501-5p), and the others showing opposing dysregulation (hsa-miR-610, hsa-miR-3168, hsa-miR-6515-3p, hsa-miR-6791-5p, hsa-piR-32608 (DQ582496). However, of those showing common directional dysregulation, only hsa-miR-148a-3p and hsa-miR-1247-3p showed expression levels above 100 reads.

### 3.2. Confirmation of ncRNA Dysregulation from RNA-seq Data with RT-qPCR in Serum and CSF

We next sought to confirm the dysregulation of candidate ncRNA in the serum of the PSP and control samples. Using the TaqMan Advanced miRNA assay chemistry, we first aimed to identify potential normalisers that showed consistent and correlated expression using NormFinder on our RNA-seq data. This helped to identify hsa-miR-148-5p, hsa-miR-191-5p, hsa-miR-5189-3p, and hsa-miR-6754-5p. Of these, when tested with RT-qPCR, only hsa-miR-191-5p showed stable expression, with a coefficient of variation of 5.6% and an average Cq of 26.4. For CSF, NormFinder identified hsa-miR-148-5p, and hsa-miR-155-5p, and hsa-miR-379-5p. Of these, hsa-miR-155-5p showed the most stable expression, with a coefficient of variation of 10.1% and an average Cq of 32.7. Attempts to find other normalisers demonstrated higher variation and/or lower expression. Based on significant dysregulation in the RNA and robust expression across all samples, we investigated expression of a total of 20 ncRNA using RT-qPCR across serum and CSF.

**Figure 2:**
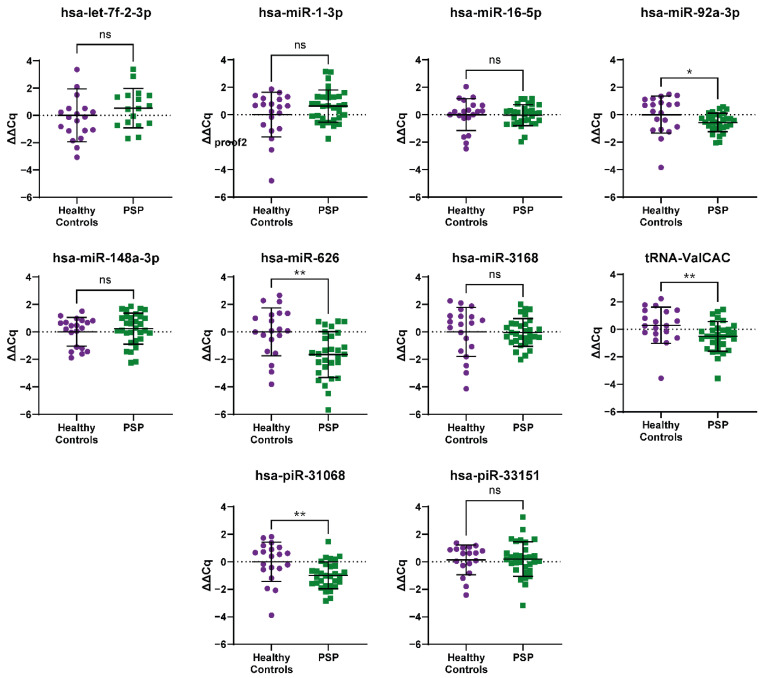
RT-qPCR profiling of ncRNA biomarker candidates in serum from RNA-seq data in PSP samples. Four of the ten ncRNA transcripts that showed normal amplification during RT-qPCR across a majority of samples showed significant dysregulation in the PSP samples compared to the average of the healthy control samples. Relative expression (control minus PSP) ± SD; normalised to hsa-miR-191-5p; hsa-piR-31068, hsa-piR-33151, hsa-miR-1-3p, hsa-miR-92a-3p, tRNA-ValCAC: Mann–Whitney test; hsa-miR-16-5p, hsa-miR-3168: Welch’s test; hsa-let-7f-2-3p, hsa-miR-148-3p, hsa-miR-626: unpaired *t*-test; * *p* < 0.05, ** *p* < 0.01, ns = not significant.

In serum, of the ten ncRNA that showed robust amplification across all samples, we found that there were significant down-regulation in hsa-miR-92a-3p (fold change (FC): -1.48), hsa-miR-626 (FC: −3.15), hsa-piR-31068 (DQ570956; FC: −1.98), and tRNA-ValCAC (FC: −1.43; Figure 2). Interestingly, hsa-miR-626, the only ncRNA in our dataset previously linked to PSP, was found to be up-regulated in our RNA-seq, the opposite of what we found with the RT-qPCR. Additionally, miR-92a-3p and hsa-piR-31068 showed significant dysregulation in the serum with RT-qPCR, despite being identified as significantly changed in the CSF only by RNA-seq, though the serum RNA-seq showed a trend of decreased expression for these ncRNA. Only the dysregulation of tRNA-ValCAC detected in the RNA-seq was confirmed with RT-qPCR in the same direction. Five other targets in serum with robust amplification, hsa-let-7f-2-3p, hsa-miR-1-3p, hsa-miR-16-5p, hsa-miR-148-3p, hsa-miR-3168, and hsa-piR-33151 were all found not to be significantly dysregulated. Additionally, hsa-miR-34a-5p, hsa-miR-206, hsa-miR-1247-3p and hsa-miR-4707-3p were tried but did not show robust amplification across all the serum samples.

Bioinformatic analysis of the potential targets of hsa-miR-92a-3p and hsa-miR-626 was carried out using miRPath for targets predicted by TargetScan and microT-CDS. Extracellular matrix-receptor interaction (*p* = 0.03, 8 genes) and regulation of actin cytoskeleton (*p* = 0.03, 21 genes) were identified as enriched KEGG pathways for hsa-miR-92a-3p, while for hsa-miR-626, enriched pathways include the epidermal growth factor receptor pathway (*p* = 0.01, 7 genes), oestrogen signalling pathway (*p* = 0.03, 7 genes) and the phosphatidylinositol signalling system (*p* = 0.03, 4 genes), in addition to cancer pathways.

**Figure 3:**
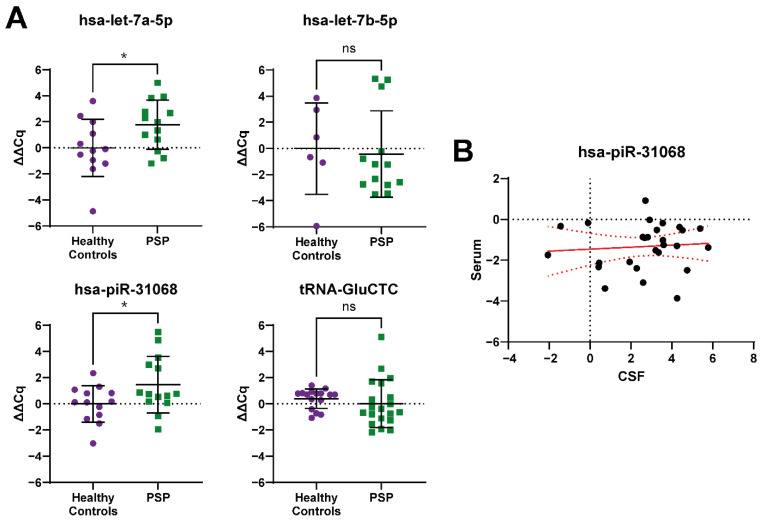
RT-qPCR profiling of ncRNA biomarker candidates in CSF from RNA-seq data in PSP samples. (**A**) Two of the four ncRNA transcripts that showed normal amplification during RT-qPCR across a majority of samples showed significant dysregulation in the PSP samples compared to the average of the healthy control samples. (**B**) No significant correlation exists between the expression of hsa-piR-30168 between serum and CSF within the same PSP patients. Relative expression (control minus PSP) ± SD; normalised to hsa-miR-155-5p; hsa-let-7b-5p, tRNA-GluCTC: Mann–Whitney test; hsa-let-7a-5p: Welch’s test; hsa-piR-31068: unpaired *t*-test; * *p* < 0.05; correlation: Bayesian correlation, ns = not significant.

In CSF, of the four that showed amplification, but not all the samples, only two of the ncRNA showed significant dysregulation with RT-qPCR. Both hsa-let-7a-5p (FC: 3.44) and hsa-piR-31068 (FC: 2.76) showed significant up-regulation, consistent with their changes observed in the RNA-seq results (Figure 3A). For hsa-piR-31068, this dysregulation was in an opposite direction to that observed in the serum above, but there are indications that there was no correlation in the expression of the piRNA between the matched serum and CSF samples (r_p_ (27) = 0.08, BF_10_ = 0.259; Figure 3B). Two other ncRNA, hsa-let-7b-5p, and tRNA-GluCTC showed no significant dysregulation. In addition, hsa-miR-92a-3p, hsa-miR-148a-3p, hsa-miR-184, hsa-miR-323b-3p, hsa-miR-486-5p, hsa-miR-1247-3p, hsa-miR-3168, and hsa-miR-4707-3p were tested but showed no amplification. Bioinformatic analysis of the potential targets of hsa-let-7a-5p using miRPath showed the top 10 KEGG pathways within which genes were enriched include cell cycle (*p* = 7.49 × 10^−9^, 43 genes), lysine degradation (*p* = 7.64 × 10^−8^, 15 genes), hippo signalling pathway (*p* = 1.37 × 10^−7^, 43 genes), oocyte meiosis (*p* = 1.98 × 10^−7^, 36 genes), extracellular matrix-receptor interaction (*p* = 4.54 × 10^−6^, 16 genes), adherens junctions (*p* = 8.15 × 10^−6^, 28 genes), and thyroid hormone signalling pathways (*p* = 6.17 × 10^−5^, 34 genes), in addition to cancer pathways.

## 4. Discussion

**Figure 4:**
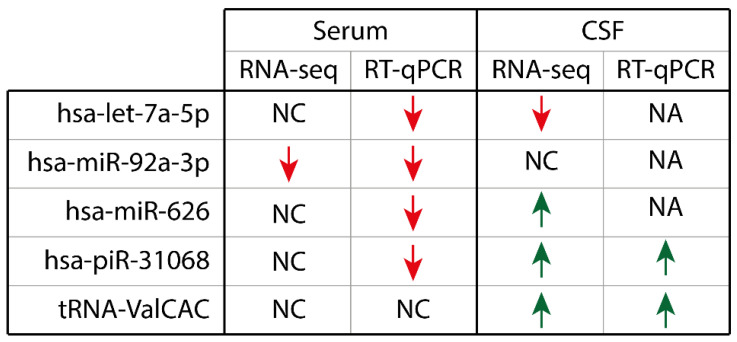
Summary of the dysregulation detected in the serum and CSF in patients with PSP compared to healthy controls. NC: no change, NA: no amplification. Red down arrow = Down-regulation, Green up arrow = Up-regulation.

Using RNA-seq, we sought to identify whether ncRNA in the serum and CSF of patients with PSP differed from controls and provide insight into the disease pathology. While we were able to identify changes using RNA-seq in both biofluids, there was limited confirmation of their dysregulation in PSP samples when RT-qPCR was undertaken. Nonetheless, we found in serum significant down-regulation of hsa-miR-92a-3p, hsa-miR-626, hsa-piR-31068, and tRNA-ValCAC while in CSF, both hsa-let-7a-5p and hsa-piR-31068 showed significant up-regulation (Figure 4). This provides evidence to suggest that there may be potential for ncRNA-based biomarkers of PSP.

One ncRNA, hsa-piR-31068, was commonly dysregulated in both serum and CSF, but it showed dysregulation in opposite directions in the two biofluids. Additionally, the lack of a correlation between its expression in the biofluids in matched PSP samples suggests that it is likely that the increase in hsa-piR-31068 in CSF is not functionally linked to the decrease in serum. Nonetheless, it is interesting that this piRNA is differentially expressed across these two biofluids, especially considering that very few ncRNA were commonly regulated in both biofluids consistent with our ALS work [27]. The function of hsa-piR-31068 is unknown, as is most piRNA outside of the gonads, but it was found to correlate with a clinical factor in pulmonary hypertension [28]. Interestingly, it is possible that this ncRNA has been misaligned in our RNA-seq, as it has been observed that the sequence is nearly identical to the sequence of tRNA fragments derived from tRNA-Gly [29]. It is difficult to know whether the original transcript is a tRNA or piRNA without further investigation, but this may explain the discord observed in the RNA-seq data between serum and CSF. Nonetheless, its detection and therefore utility as a biomarker for PSP is noteworthy.

Another one of our candidates that shows promise is the tRNA 5′ fragment derived from tRNA-ValCAC, which was the only one of the four ncRNA in serum that showed down-regulation with RT-qPCR consistent with the RNA-seq. The function of tRNA fragments is still relatively unknown but they can be actively cleaved by angiogenin, have been shown to affect RNA translation and be induced by stress [30,31]. In fact, tRNA fragments have been proposed previously as biomarkers for ALS, including in our own studies [18,32]. Most interestingly though, one study, found that 5′ fragments derived from tRNA-ValCAC had elevated release from neural cells and cleavage in mouse models, and consequently had prognostic value when tested in human samples [32]. This may suggest that while this marker may not be specific for PSP, it could still be of value in conjunction with other markers.

One area of concern is the discord between our RNA-seq and RT-qPCR results. For example, hsa-miR-626 was found up-regulated in the RNA-seq but down-regulated in the RT-qPCR. However, hsa-miR-626 has been previously suggested to be possibly useful in classifying PSP samples in comparison with PD [15]. Additionally, RNA-seq did not detect the significant dysregulation of hsa-miR-92a-3p in serum shown with RT-qPCR, but it did detect changes in CSF, though attempts to profile it in CSF using RT-qPCR were unsuccessful. However, the discord between the direction of dysregulation is concerning, with no evident reason why this may have occurred. This may have been due to the low annotation rates for ncRNA in serum and CSF, even though our results are consistent with other studies including our own previous studies where we were more successful [18,27,33,34,35]. However, in CSF, we were slightly more successful with two ncRNA that validated their dysregulation in the same direction. In addition to hsa-piR-31068, hsa-let-7a-5p showed significant up-regulation in PSP samples. This miRNA has also been linked to PD as a potential biomarker in plasma [36], but its potential function or role in PSP pathology is unknown. Indeed, how these ncRNA contribute to the pathology of PSP is unknown as they have not previously been linked to the disease. While bioinformatic analysis was undertaken to identify target pathways, this is of limited use as the target or source tissues of these ncRNA is unknown. Additionally, the function of these ncRNA in the serum or CSF is also unknown, as to whether they are part of the paracrine signalling system between cells or present as a waste product from dying cells. As such, it is difficult to attribute how these ncRNA may be involved in the pathology of PSP, but future studies investigating ncRNA in post-mortem human tissue may help provide more clarity as to their role.

Two recent studies have been published investigating microRNA as biomarkers in plasma and CSF from patients with PSP. In plasma, profiling of miRNA in 18 patients with PSP and 17 healthy controls using RT-qPCR arrays found that miR-19b-3p, miR-33a-5p, miR-130b-3p, miR-136-3p, and miR-210-3p showed potential as biomarkers for PSP [37]. Another recent study investigating the CSF of 11 patients with PSP and 8 healthy controls showed changes in miR-204-3p, miR-873-3p and miR-6840-5p with the progression of the disease [38]. However, these eight markers identified in the previous studies were not changed in our RNA-seq data. This is consistent with the variation in dysregulated ncRNA that we have seen in biomarker studies in ALS and in part is likely due to variations in sample type, handling, and analysis [39]. While a meta-analysis of these three studies to increase the number and diversity of the samples analysed would be limited, future studies should increase sample size with samples across multiple cohorts to address these limitations.

One thing that must be considered as with all studies investigating CSF as a potential source of ncRNA-based biomarkers is whether it is a suitable biofluid for diagnosis. The majority of targets that we attempted to measure in CSF were unsuccessful due to poor amplification with RT-qPCR, reflecting low amounts present in CSF despite only choosing those with robust expression levels. Bearing these data in mind and considering the invasive nature of collecting CSF, this biofluid may not be the most appropriate source of circulating ncRNA. With a more routine collection process, serum derived from blood may be a more suitable source for diagnostic markers, and future work should be focussed on identifying potential candidates within it. Combined with having identified potential candidates to be biomarkers for PSP, serum may be a more prudent source for further investigation. However, while we have identified potential candidates, the disconnect between the RNA-seq data and the RT-qPCR raises the possibility that there are other suitable candidates that may not have been identified and that a study with increased patient samples would be required. This would also allow for identification and testing of discriminating models like binomial logistic regression or random forest models using the expression of the biomarkers to classify samples. The small size of the current cohort limits the power of such analysis but with increased sample size, such analysis could be powerful and allow these ncRNA to be used despite the overlap in expression between the PSP and control samples. Nonetheless, this study has shown that there may be potential dysregulated ncRNA in biofluids that could be used to aid in diagnosis and elucidate pathogenic mechanisms of PSP.

## 5. Methods

### 5.1. Patient Information

**Figure 5:**
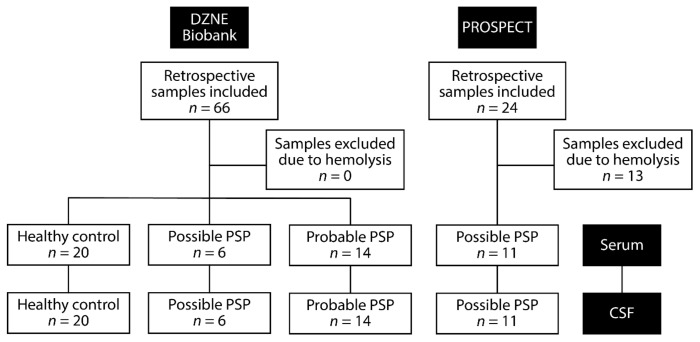
Flowchart of samples used in study.

Two sets of samples were utilised as part of this study (Figure 5, Table 1). Firstly, samples used for the RNA-seq screen were collected as part of the Biobank of the German Center of Neurodegenerative Diseases (DZNE e.V.) in Bonn, Germany. Ethical approval for the extraction and use of the biofluid samples and associated clinical data for this study were obtained from the Ethics Committee of the Faculty of Medicine of the Technical University of Munich. All participants provided written consent (or gave permission for a carer to sign on their behalf). This cohort comprised of 20 PSP patients as diagnosed to the MDS-PSP criteria [4], and 20 age- and sex-matched controls with no history or clinical signs of neurodegenerative disease.

We also obtained additional PSP samples from the Progressive Supranuclear Palsy–Corticobasal Syndrome–Multiple System Atrophy (PROSPECT) study in the UK [6]. Ethical approval for sample collection and their use was approved by UCL Queen Square Institute of Neurology research ethics committee, with all recruited participants providing written informed consent. Patients were defined as PSP using the NINDS-SPSP criteria [3], which closely corresponds to probable PSP-Richardson’s syndrome under the MDS 2017 criteria [4].

### 5.2. Sample Collection, Preparation, and RNA Extraction

Matched serum and CSF samples were obtained from each PSP patient and healthy control. For serum samples, blood was collected from patients into BD Vacutainer SST tubes, left to clot at room temperature and centrifuged at 3000 rpm for 10 min at 4˚C. The serum supernatant was then removed and aliquoted into 1.8 mL aliquots and stored at -80˚C. Minimal red blood cell lysis was checked using a haemoglobin ELISA (ab157707, Abcam) with a threshold of 0.6 g/L [40]—13 additional samples originally received from the PROSPECT study exceeded this threshold and were excluded from those used in the study. Small RNA was isolated from a 200 µL sub-aliquot of individual serum samples using the miRNeasy Micro kit (Qiagen) with a DNase I treatment (Qiagen).

CSF samples were obtained by lumbar puncture directly into polypropylene collection tubes. Samples were centrifuged at 3000 rpm for 10 min at 4 °C within 1 h of sampling and stored at −80 °C until extraction. CSF samples were checked for any contamination for blood by visual inspection. Small RNA was isolated from a 400 µL sub-aliquot of individual CSF samples using the miRNeasy Micro kit (Qiagen) with a DNase I treatment (Qiagen).

### 5.3. RNA-Sequencing

Samples from the healthy controls, possible PSP and probable PSP groups from the DZNE cohort were subdivided into pools of five samples each. For serum, for each pool, 1 ng of extracted small RNA per sample as quantified by the Small RNA Bioanalyser kit (Agilent) were combined. For the CSF, for each pool, fixed and equal volumes 7 µL of extracted RNA per sample from CSF were combined. These pools were then concentrated using a SpeedVac at ambient temperature for 40 min from 50 µL to 8 µL. Each sample pool was then converted into RNA-Seq libraries using the QIAseq miRNA library kit (Qiagen) following the recommended parameters for serum RNA. These libraries then underwent 75 bp paired-end sequencing on an Illumina NextSeq machine at the University of Leeds, United Kingdom. Data were pre-processed to remove 5′ and 3′ adaptors and then underwent two analysis pipelines. The automated Qiagen QIASeq miRNA pipeline read the unique molecular indexes (UMI), sequentially aligned the RNA-seq data to a database of ncRNA transcripts using Bowtie. Reads from the analysis were then normalised and differential expression calculated between sample groups using DESeq2 [41].

### 5.4. RT-qPCR Confirmation and Analysis

Using the TaqMan Advanced miRNA RT-qPCR chemistry (Applied Biosystems), a fixed volume of 2 µL of RNA extracted from all samples was converted to cDNA with pre-amplification and diluted ten-fold. Using pre-designed primers and master mixes (Applied Biosystems), candidate transcripts were quantified using fast cycling conditions on a QuantStudio 7 cycling machine. Pre-designed miRNA primers used included hsa-miR-1-3p (477820_mir), hsa-let-7a-5p (478575_mir), hsa-let-7b-5p (478576_mir), hsa-let-7f-2-3p (477843_mir), hsa-miR-16-5p (477860_mir), hsa-miR-34a-5p (478048_mir), hsa-miR-92a-3p (477827_mir), hsa-miR-148-3p (478718_mir), hsa-miR-148a-5p (478718_mir), hsa-miR-155-5p (483064_mir), hsa-miR-184 (477938_mir), hsa-miR-191-5p (477952_mir), hsa-miR-206 (477968_mir), hsa-miR-486-5p (479128_mir), hsa-miR-626 (479110_mir), hsa-miR-1247-3p (479553_mir), hsa-miR-3168 (480795_mir), hsa-miR-4707-3p (479946_mir), hsa-miR-5189-3p (480103_mir), and hsa-miR-6754-5p (480280_mir) with primers against tRNA and piRNA custom designed. Cq values were averaged and normalised to the hsa-miR-191-5p for serum and hsa-miR-155-5p for CSF, which were identified using NormFinder and confirmed with RT-qPCR [42]. An average for the healthy control samples was calculated and all samples were compared to this average for a ΔΔCq value. To aid in interpretation of the data, relative expression was calculated with control minus PSP to provide ΔΔCq values where a decrease in ΔΔCq represents a decrease in expression in the PSP group and vice versa.

### 5.5. Bioinformatic Analysis of MicroRNA Targets

Targets of validated miRNA were identified using the preloaded lists of microT-CDS and TargetScan on mirPath 3.0 and used to identify enriched KEGG pathways [43].

### 5.6. Statistical Analysis

Statistical analyses for RT-qPCR were conducted on ΔΔCq values for each sample with GraphPad Prism 8.0. Outliers were identified using the ROUT method in GraphPad Prism 8.0 (Q = 1%). Distribution of the data were determined using a Shapiro–Wilks normality test. One-way ANOVA was carried out across the four groups with Tukey’s multiple comparison for parametric data and a Welch’s one-way ANOVA with Gomes-Howell multiple comparison when nonparametric. All statistics were two-tailed and significance was set at *p* < 0.05. For correlation analysis, Bayesian correlation was undertaken in JASP, with a BF_10_ > 1 providing evidence for a correlation, and BF_10_ < 1 providing evidence against a correlation.

## Figures and Tables

**Table 1:** Characteristics of the cohorts whose samples were used in this study. M/F: male/female.

	DZNE Biobank			PROSPECT
	Healthy Controls	Possible PSP	Probable PSP	Possible PSP
**Number of Patients**	20	6	14	11
**Sex (M/F)**	9/11	6/0	10/4	9/2
**Average age at sampling (year)**	64.2 (30–79)	69.8 (64–75)	72.4 (66–82)	69.5 (59–81)

## Data Availability

The data that support the findings of this study have been deposited onto Sequence Read Archive under accession PRJNA809921 (https://www.ncbi.nlm.nih.gov/bioproject/PRJNA809921; accessed on 13 November 2022).

## Abbreviations

4R-tau: 4-repeat Tau; AUC: area under curve; CA: classification accuracy; Cq: quantitative cycle; CSF: cerebrospinal fluid; ncRNA: non-coding RNA; miRNA: microRNA; piRNA: piwi-interacting RNA; PE: paired end; RNA-seq: RNA-sequencing; rRNA: ribosomal RNA; RT-qPCR: reverse transcription-quantitative PCR; snoRNA: small nucleolar RNA; snRNA: small nuclear RNA; tRNA: transfer RNA; UMI: unique molecular indexes.
